# Air Contamination by Mercury, Emissions and Transformations—a Review

**DOI:** 10.1007/s11270-017-3311-y

**Published:** 2017-03-03

**Authors:** Barbara Gworek, Wojciech Dmuchowski, Aneta H. Baczewska, Paulina Brągoszewska, Olga Bemowska-Kałabun, Justyna Wrzosek-Jakubowska

**Affiliations:** 1 Institute of Environmental Protection-National Research Institute, Krucza 5/11d St., Warsaw, 00-548 Poland; 2 Department of Agriculture and Biology, Warsaw University of Life Sciences, Nowoursynowska 159 St., Warsaw, 02-776 Poland; 3 Polish Academy of Sciences, Botanical Garden - Center for Biological Diversity Conservation, Prawdziwka 2 St., 02-973 Warsaw, Poland

**Keywords:** Mercury, Air pollution, Emission, Transformation

## Abstract

The present and future air contamination by mercury is and will continue to be a serious risk for human health. This publication presents a review of the literature dealing with the issues related to air contamination by mercury and its transformations as well as its natural and anthropogenic emissions. The assessment of mercury emissions into the air poses serious methodological problems. It is particularly difficult to distinguish between natural and anthropogenic emissions and re-emissions from lands and oceans, including past emissions. At present, the largest emission sources include fuel combustion, mainly that of coal, and “artisanal and small-scale gold mining” (ASGM). The distinctly highest emissions can be found in South and South-East Asia, accounting for 45% of the global emissions. The emissions of natural origin and re-emissions are estimated at 45–66% of the global emissions, with the largest part of emissions originating in the oceans. Forecasts on the future emission levels are not unambiguous; however, most forecasts do not provide for reductions in emissions. Ninety-five percent of mercury occurring in the air is Hg^0^—GEM, and its residence time in the air is estimated at 6 to 18 months. The residence times of its Hg^II^—GOM and that in Hg_p_—TPM are estimated at hours and days. The highest mercury concentrations in the air can be found in the areas of mercury mines and those of ASGM. Since 1980 when it reached its maximum, the global background mercury concentration in the air has remained at a relatively constant level.

## Introduction

Mercury (Hg) is one of the most important trace elements emitted to the atmosphere due to its toxic effects on the environmental and human health, as well as its role in the chemistry of the atmosphere and other environmental compartments and global atmospheric transport with air masses (Pacyna and Pacyna 2002). Hg is an atmospheric pollutant with a complex biogeochemical cycle. The atmospheric cycling includes chemical oxidation/reduction in both gaseous and aqueous phases, deposition and re-emission from natural surfaces in addition to emissions from both natural and anthropogenic sources (Wängberg et al. 2001). The toxicity of Hg and its compounds for humans such as ataxia, constriction of vision, impaired hearing and death was first described in 1865 (Grandjean et al. 2010). In 2009, the Governing Council of the United Nations Environment Programme (UNEP) began development of a legally binding global instrument on Hg. In 2013, governments agreed to text for this instrument, thus giving birth to the Minamata Convention on Mercury. Convention has been signed by more than 120 nations and is now being ratified. The primary objective of the convention is to “protect human health and the environment from anthropogenic emissions and releases of Hg and Hg compounds” (UNEP Minamata Convention 2014).

## Mercury Transformations in the Air

Mercury can occur in the atmospheric environment in different species. In the literature, the following terminologies have been adopted to enable the identification of its different chemical and physical forms and compounds (Lindberg et al. 2002):

- THg (total mercury) the sum of all mercury species
- TGM (total gaseous mercury) the sum of all gaseous compounds and gaseous elemental mercury (Hg^0^)
- GEM (gaseous elemental mercury) gaseous elemental mercury (Hg^0^)
- GOM (gaseous oxidised mercury) gaseous mercury in oxidised form
- RGM (reactive gaseous mercury) reactive gaseous mercury, chemical compounds of the oxidized form of divalent mercury (Hg^II^)
- TPM or Hg_p_ (total particulate mercury) all mercury compounds contained in particulate matter of different, unspecified chemical form and particulate size
- PBM (particle-bound mercury) mercury compounds contained in particulate matter
- MeHg—organic mercury compounds

The concentration of mercury in the air depends on the degree of volatility of its compounds which is strictly related to the ambient temperature. The quantity of evaporated Hg^0^ doubles for each temperature increase of 10 °C. The volatility of compounds falls in the following (decreasing) order: Hg^0^ > Hg_2_Cl_2_ > HgCl_2_ > HgS > HgO (Kabata-Pendias and Pendias 2001).

Hg^0^ is characterised by much higher levels of vapour pressure levels than the compounds of Hg^II^ in the second oxidation state. At a temperature of 25 °C it is 0.2 pa for Hg^0^ and 0.1 pa for Hg^II^ (Lindquist et al. 1991; Rayaboshapko and Korolev 1997).

GOM represents about 98% of the mass of mercury which is present in the air. It occurs in three oxidation states: Hg^0^, Hg^I^ and Hg^II^. GEM dominates, representing about 95% of its total mass, the second oxidation state (Hg^II^) occurs in small amounts, while the first oxidation state (Hg^+^) can be found in trace amounts (Schroeder et al. 1998).

The following abbreviations are used in the reactions presented below: (g)—the gaseous state, (s)—the solid state, (aq)—the liquid state. Oxidation of the gaseous species GEM is the most important process of mercury removal from the air. The main oxidation reaction is that of GEM with ozone (O_3_) (Schroeder et al. 1998; Biswajit and Parisa 2003; Lin et al. 2012). Tropospheric O_3_ is a secondary air pollutant arising as a result of photochemical reactions (Hall 1995; Finlayson-Pitts and Pitts 2000).

$$ {\mathrm{Hg}}^0\left(\mathrm{g}\right)+{\mathrm{O}}_3\left(\mathrm{g}\right)\to \mathrm{H}\mathrm{g}\mathrm{O}\left(\mathrm{s}\right)+{\mathrm{O}}_2\left(\mathrm{g}\right) $$

The oxidation of GEM by the hydroxyl radical (^**•**^OH) also contributes to the removal of substantial quantities of mercury from the atmosphere (Lin et al. 2012). Hydroxyl radicals in the air are generated by the reactions of water vapour with atomic oxygen arising from the photolysis of O_3_, nitrogen oxides (NO_*x*_) and hydrogen peroxide (H_2_O_2_) (Goodsite et al. 2004; van Loon and Duffy 2005).

$$ \begin{array}{l}{\mathrm{Hg}}^0\left(\mathrm{g}\right){+}^{\bullet}\mathrm{OH}\left(\mathrm{g}\right){\to}^{\bullet}\mathrm{H}\mathrm{gO}\mathrm{H}\left(\mathrm{g}\right)\hfill \\ {}{}^{\bullet}\mathrm{H}\mathrm{gO}\mathrm{H}\left(\mathrm{g}\right)+{\mathrm{O}}_2\left(\mathrm{g}\right){\to}^{\bullet}\mathrm{H}\mathrm{gO}\left(\mathrm{s}\right){+}^{\bullet}\mathrm{OH}\left(\mathrm{g}\right)\hfill \end{array} $$

The oxidation of GEM by the nitrate radical (^•^NO_3_) also plays a certain role in the removal of mercury from the atmospheric air. The radical is primarily generated by the reaction between O_3_ and nitrogen dioxide (NO_2_), which mainly takes place in night-time and, to a lesser extent, by the photolysis of nitrogen pentoxide (N_2_O_5_), which unfolds in daytime (Finlayson-Pitts and Pitts 2000; Lindberg et al. 2002; Lin et al. 2012).

$$ {\mathrm{Hg}}^0\left(\mathrm{g}\right){+}^{\bullet }{\mathrm{NO}}_3\left(\mathrm{g}\right)\to \mathrm{H}\mathrm{g}\mathrm{O}\left(\mathrm{s}\right)+{\mathrm{NO}}_2\left(\mathrm{g}\right) $$

GEM also reacts in the air with the atoms, compounds and radicals of chlorine, bromine and iodine. They occur mainly in sea salt aerosols, particularly in coastal areas (Goodsite et al. 2004; Lin et al. 2012).

$$ \begin{array}{l}{\mathrm{Hg}}^0\left(\mathrm{g}\right)+\mathrm{Cl}\left(\mathrm{g}\right)\to \mathrm{HgCl}\left(\mathrm{g}\right)\hfill \\ {}{\mathrm{Hg}}^0\left(\mathrm{g}\right)+\mathrm{Br}\left(\mathrm{g}\right)\to \mathrm{HgBr}\left(\mathrm{g}\right)\hfill \\ {}\mathrm{Hg}(0)\left(\mathrm{g}\right)+\mathrm{F}\left(\mathrm{g}\right)\to \mathrm{HgF}\left(\mathrm{g}\right)\hfill \\ {}{\mathrm{Hg}}^0\left(\mathrm{g}\right)+\mathrm{I}\left(\mathrm{g}\right)\to \mathrm{HgI}\left(\mathrm{g}\right)\hfill \\ {}{\mathrm{Hg}}^0\left(\mathrm{g}\right)+{\mathrm{Cl}}_2\left(\mathrm{g}\right)\to {\mathrm{Hg}\mathrm{Cl}}_2\left(\mathrm{g}\right)\hfill \\ {}{\mathrm{Hg}}^0\left(\mathrm{g}\right)+{\mathrm{Br}}_2\left(\mathrm{g}\right)\to {\mathrm{Hg}\mathrm{Br}}_2\left(\mathrm{g}\right)\hfill \\ {}{\mathrm{Hg}}^0\left(\mathrm{g}\right)+{\mathrm{F}}_2\left(\mathrm{g}\right)\to {\mathrm{Hg}\mathrm{F}}_2\left(\mathrm{g}\right)\hfill \\ {}{\mathrm{Hg}}^0\left(\mathrm{g}\right)+\mathrm{Cl}\mathrm{O}\left(\mathrm{g}\right)\to \mathrm{HgCl}\mathrm{O}\left(\mathrm{g}\right)\hfill \\ {}{\mathrm{Hg}}^0\left(\mathrm{g}\right)+\mathrm{Br}\mathrm{O}\left(\mathrm{g}\right)\to \mathrm{HgBr}\mathrm{O}\left(\mathrm{g}\right)\hfill \end{array} $$

RGM occurs in the air in very low concentrations (pg/m^3^) and because of low vapour pressures it very quickly undergoes wet deposition to the surface (Schroeder et al. 1998; Mason 2005; Mason et al. 2010).

Mercury also occurs in the atmospheric air in the form of methyl compounds, mainly dimethylmercury (CH_3_)_2_Hg^0^). It is an inert, hardly soluble and volatile compound (van Loon and Duffy 2005). The share of mercury in organic compounds represents only 0.3–1.0% of the total amount of mercury in the air (Lee et al. 2003). Examples of reactions are shown below (Niki et al. 1983a, b):

$$ \begin{array}{l}{\left({\mathrm{CH}}_3\right)}_2\mathrm{Hg}\left(\mathrm{g}\right)+{\mathrm{O}}_3\left(\mathrm{g}\right)\to \mathrm{products}\hfill \\ {}{\left({\mathrm{CH}}_3\right)}_2\mathrm{Hg}\left(\mathrm{g}\right){+}^{\bullet}\mathrm{OH}\left(\mathrm{g}\right)\to {\left({\mathrm{CH}}_3\right)}_2\mathrm{Hg}\mathrm{OH}\left(\mathrm{g}\right){+}^{\bullet }{\mathrm{CH}}_3\hfill \\ {}{\left({\mathrm{CH}}_3\right)}_2\mathrm{Hg}\left(\mathrm{g}\right)+{\mathrm{Cl}}^{\bullet}\left(\mathrm{g}\right)\to {\mathrm{CH}}_3\mathrm{HgCl}\left(\mathrm{g}\right){+}^{\bullet }{\mathrm{CH}}_3\left(\mathrm{g}\right)\hfill \\ {}{\left({\mathrm{CH}}_3\right)}_2\mathrm{Hg}\left(\mathrm{g}\right){+}^{\bullet }{\mathrm{NO}}_3\left(\mathrm{g}\right)\to {\mathrm{Hg}}^0\left(\mathrm{g}\right)+\mathrm{HgO}\left(\mathrm{s}\right)+\mathrm{other}\ \mathrm{products}\hfill \end{array} $$

Mercury also occurs in different forms in the water phase: in raindrops, fog and clouds. The type of its transformations depends on temperature, insolation, reaction and other pollutants. Hg^0^ dissolved in water may undergo oxidation and, as a rule, the end product of these reactions is the ionic form Hg^2+^, e.g. (Lin et al. 2012):

$$ \begin{array}{l}{\mathrm{H}}^{+}\hfill \\ {}{\mathrm{H}\mathrm{g}}^0\left(\mathrm{aq}\right)+{\mathrm{O}}_3\left(\mathrm{aq}\right)\to {\mathrm{H}\mathrm{g}}^{2+}\left(\mathrm{aq}\right)+\mathrm{OH}\hbox{-} \left(\mathrm{aq}\right)+{\mathrm{O}}_2\left(\mathrm{aq}\right)\hfill \\ {}{\mathrm{H}\mathrm{g}}^0\left(\mathrm{g}\right){+}^{\bullet}\mathrm{OH}\left(\mathrm{g}\right)\to {\mathrm{H}\mathrm{g}}^{2+}\left(\mathrm{aq}\right)+\mathrm{other}\ \mathrm{products}\hfill \\ {}{\mathrm{H}\mathrm{g}}^0\left(\mathrm{aq}\right)+\mathrm{HOCl}\left(\mathrm{aq}\right)\to {\mathrm{H}\mathrm{g}}^{2+}\left(\mathrm{aq}\right)+\mathrm{OH}\hbox{-} \left(\mathrm{aq}\right)+\mathrm{Cl}\hbox{-} \left(\mathrm{aq}\right)\hfill \\ {}{\mathrm{H}\mathrm{g}}^0\left(\mathrm{aq}\right)+\mathrm{HOBr}\left(\mathrm{aq}\right)\to {\mathrm{H}\mathrm{g}}^{2+}\left(\mathrm{aq}\right)+\mathrm{OH}\hbox{-} \left(\mathrm{aq}\right)+\mathrm{Br}\hbox{-} \left(\mathrm{aq}\right)\hfill \end{array} $$

Examples of reactions are shown below. In some cases, these are photochemical reactions (Lin et al. 2012):

$$ \begin{array}{c}\hfill {}_{hv}\hfill \\ {}\hfill \mathrm{Hg}{\left(\mathrm{OH}\right)}_2\left(\mathrm{aq}\right)\to \mathrm{Hg}\mathrm{OH}\left(\mathrm{aq}\right){+}^{\bullet}\mathrm{OH}\left(\mathrm{aq}\right)\hfill \\ {}\hfill {}_{hv}\hfill \\ {}\hfill \mathrm{Hg}{\left(\mathrm{OH}\right)}_2\left(\mathrm{aq}\right)\to {\mathrm{Hg}}^0\left(\mathrm{aq}\right)+\mathrm{other}\ \mathrm{products}\hfill \\ {}\hfill {\mathrm{Hg}}^{2+}\left(\mathrm{aq}\right)+{\mathrm{HO}}_2\to {\mathrm{Hg}}^0\left(\mathrm{aq}\right)+\mathrm{other}\ \mathrm{products}\hfill \\ {}\hfill \mathrm{Hg}{\left({\mathrm{SO}}_3\right)}^{2\hbox{-}}\left(\mathrm{aq}\right)\to {\mathrm{Hg}}^0\left(\mathrm{aq}\right)+\mathrm{S}\left(\mathrm{VI}\right)\hfill \end{array} $$

Henry’s law defines the amount of a gas which can be dissolved in a liquid. At a given pressure and temperature, a liquid, which is water in the present case, contains a certain amount of dissolved gases. Henry’s law constant is characteristic of a given water-gas system. The solubility of gaseous mercury in water diminishes with increasing temperature and decreasing pressure. The lower the value of Henry’s law constant is, the more gas can be dissolved in it under given conditions (Andersson et al. 2008).

υ: K_h_(T)p
K_h_(T): Henry’s law constant
p: the pressure over the surface of the liquid
υ: the volume of the gas dissolved in a unit of mass or volume of the liquid.

GEM and MeHg are characterised by incomparably lower solubility in water than inorganic compounds of Hg^II^: Hg^0^—0.12, (CH_3_)Hg—0.13, HgCl_2_—1.4 × 10^6^, Hg(OH)_2_—1.2 × 10^4^ (Seigneur et al. 1994; Rayaboshapko and Korolev 1997).

The residence time of mercury in the air depends on many factors. Apart from the weather conditions, they also include the degree and type of air pollutants. In the case of GEM, the residence time is estimated at 6 to 18 months, while GOM and TPM contained in particulate matter are quickly removed from the air through wet and dry deposition, and their residence times are estimated at most at hours and days (Selin et al. 2007; Skov et al. 2008; Mason et al. 2010).

Given the long time of its removal from the air, GEM can be transported over large distances. The path of mercury from Asia to Northern America has been particularly investigated. Travnikov et al. (2008) estimated the migration time of mercury at 5 to 10 days, with its largest amounts transported in the spring.

There are many problems related to the mercury contamination of the atmospheric air. They include episodes of sudden drops in TGM concentrations in the Antarctic and Arctic. These interesting phenomena are called MDEs (mercury depletion events). Schroeder et al. (1998) were some of the first scientists to investigate them at the scientific research station Alert in north-western Canada (82.5 N, 62.5 W). The mercury concentration in the atmospheric air decreased in the spring, from early April to mid-June. The “normal” mercury concentration in this region was 1.84 ng/m^3^, whereas in the spring, it fell to a level of much less than 1 ng/m^3^ and even to less than 0.1 ng/m^3^ in 24-h measurements. This was accompanied by an ozone pressure drop from 30 to 50 to 10 ppb and, at times, even less than 0.5 ppb. Mercury and ozone depletion events in the Arctic were demonstrated earlier by Barrie et al. (1988) and Anlauf et al. (1994).

Springtime mercury and ozone depletion events can also be observed in the Antarctic where they occur from the end of August to the end of October (Ebinghaus et al. 2002; Brooks et al. 2008; Witherow and Lyons 2008). The research carried out at the German Antarctic Station Neumayer (70° 39′ S, 8° 15′ W) demonstrated that as a result of its springtime depletion in the air, TGM fell from the “normal” level of 1.1–1.2 to 0.9 ng/m^3^, with the minimum value of 0.1 ng/m^3^ (Ebinghaus et al. 2002). The more recent research carried out by Pfaffhuber et al. (2012) at the Norwegian Antarctic Station Troll (72° 01′ S, 2° 32′ E) showed a greater difference: the GEM concentration was 1.0 ng/m^3^, whereas in the springtime mercury depletion period it was 0.6 ng/m^3^.

This phenomenon is caused by the oxidation of GEM to its GOM form. GEM is characterised by a low value of Henry’s law constant, which indicates its very low solubility in water (Skov et al. 2008; Seigneur et al. 1994; Rayaboshapko and Korolev 1997). This results in its long residence time in the air—even up to a year. However, in the Arctic conditions its residence time is estimated at 10 h only (Goodsite et al. 2004; Skov et al. 2004).

In the spring in the Arctic and Antarctic, there are favourable conditions for photochemical processes (long days) which result in higher halide concentrations. According to Ariya et al. (2004), such forms as Br and BrO, rather than Cl, Cl_2_ or Br_2_, are mainly responsible for MDEs. It is primarily bromine that participates in the mercury oxidation reaction. Bromine originates from sea aerosols under the conditions of intensive UV radiation. Bromine emissions into the air are strictly related to seawater defreezing at the sea ice temperature of less than −4 °C (Lindberg et al. 2002; Skov et al. 2008; Obrist et al. 2011). The share of bromine in MDEs is estimated at 73.9% (Calvert and Lindberg 2003). Oxidized mercury compounds are quickly deposited to the surface. Ozone also participates in this process, as it is involved in the oxidation of gaseous elemental mercury, but is also quickly decomposed by halides (Ariya 2011; Lopez et al. 2007; Holmes et al. 2010).

**Fig. 1:**
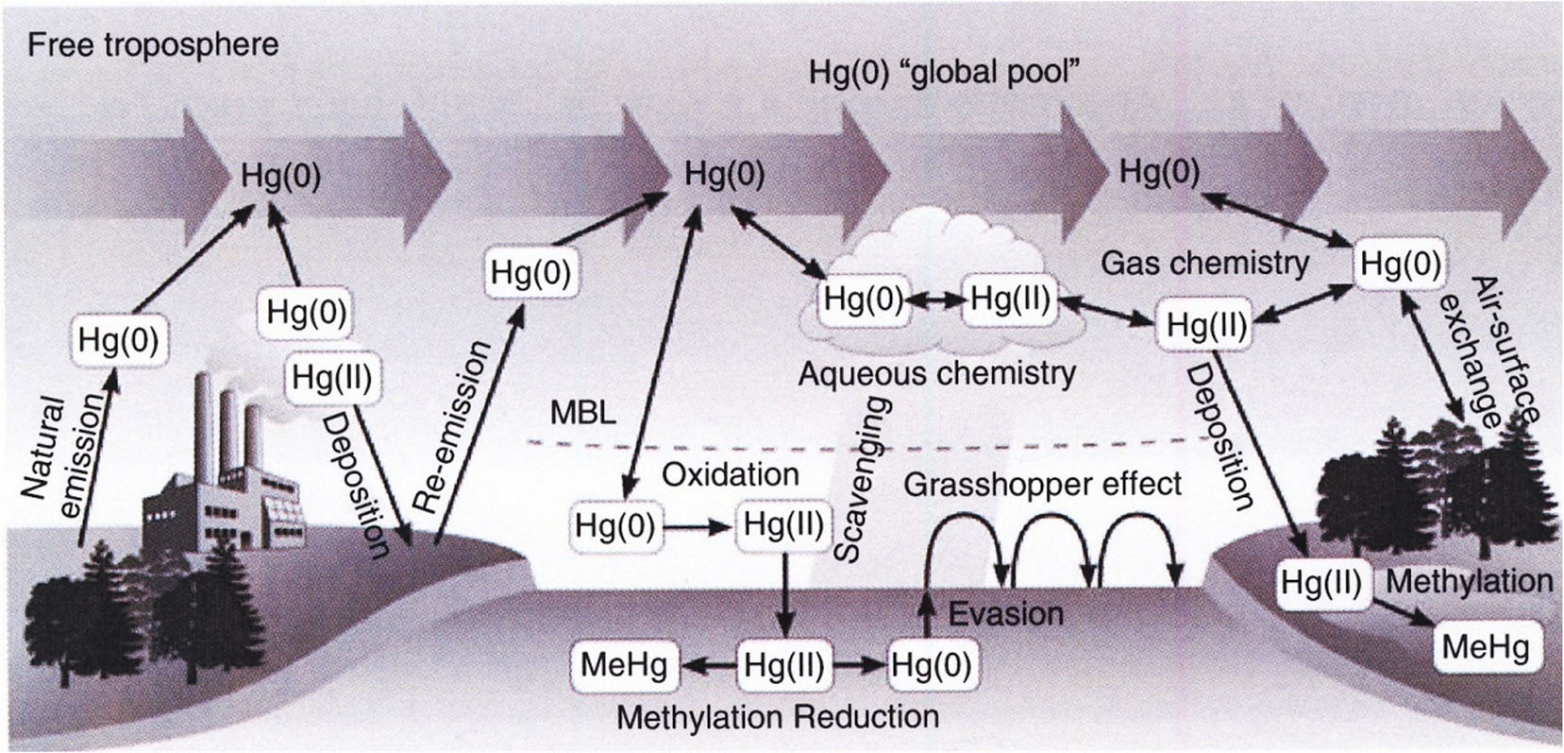
General concept of the overall Hg cycle in the atmosphere (Travnikov 2012)

General concept of the overall Hg cycle in the atmosphere is presented in the Fig. 1 (Travnikov 2012).

## Mercury Emissions into the Air

Mercury emission sources include both natural processes unfolding in the biosphere and anthropogenic sources. In 2008, the following classification was adopted in the UNEP report (2008, Global Atmospheric Mercury Assessment: Sources, Emissions and Transport), which distinguished between three emission sources:

- Current emissions from natural sources
- Current emissions from anthropogenic sources
- Re-emissions from past deposits from natural and anthropogenic sources

The assessment of mercury emissions poses serious methodological problems. In estimating them, state institutions mainly focus on inventories of their sources, while international organisations apply different models, using emission factors and statistical data on the industrial production and consumption of mercury-containing materials. It is particularly difficult to distinguish natural and anthropogenic emissions from re-emissions from lands and oceans (Pacyna et al. 2010a, b).

To a large extent, various models are applied to estimate mercury emission levels into the atmosphere. However, the emission levels determined by using them are substantially different. Travnikov et al. (2008) compared the global emission levels from natural and anthropogenic sources determined by using four models which are now applied (Table 1). Various models are applied to determine emission levels. However, the models which are probably most often used, i.e. the CTM-Hg, GEOS-Chem and GRAHM models, produced greatly different global emission levels, varying between 5700 and 9230 t/year, but these estimates kept the relative proportions between natural and anthropogenic emissions. The MSCE-HM model showed a much lower global emission level than those determined by the other models compared and was the only one to give a higher level of emissions of anthropogenic origin than those of emissions from natural sources and re-emission. The cited data indicate how imperfect model-based methods still are and how difficult and complicated research on mercury in the environment is.

**Table 1:** Global emissions of mercury to the atmosphere estimated by using various models (Mg/year)

Type of emission	CTM-Hg	GEOS-Chem	GRAHM	MSCE-HM
Anthropogenic	2200 (34%)	3400 (37%)	2200 (39%)	2200 (55%)
Natural and remission	4340 (66%)	5830 (63%)	3500 (61%)	1800 (45%)
All	6540	9230	5700	4000

*CTM-Hg* Global Chemical Transport Model for Mercury (Seigneur et al. 2009), *GEOS-Chem* Goddard Earth Observing System (Henze at al. 2006), *GRAHM* Global/Regional Atmospheric Heavy Metals (Dastoor and Larocque 2004), *MSCE-HM* Meteorological Synthesizing Centre-East (Travnikov and Ilyin 2009)

Mercury flux values are different from one another, depending on the methods applied (Table 2). In their estimates, many authors do not distinguish re-emission. It is estimated that the total annual mercury emissions into the air from all its sources exceed 5000–7000 t (Mason et al. 1994; Lamborg et al. 2002; Gray and Hines 2006). The proportions between the levels of natural and anthropogenic emissions are not determined accurately. Depending on the authors, this ratio is given in a relatively wide range of 0.8 to 1.8 (Nriagu and Pacyna 1998; Nriagu 1989, 1994; Nriagu and Becker 2003; Seigneur et al. 2004; Gbor et al. 2007; Shetty et al. 2008; Liu et al. 2012). The relatively wide emission ranges presented in different studies are caused by the instability and volatility of its compounds, the dispersion of its sources, low mercury concentrations in the air, which are much lower than those of other basic pollutants, and the difficulties related to their determination (Gustin et al. 2000, 2008).

**Table 2:** Estimating the flow of mercury in the environment (kt/year) (Wilson et al. 2012 modified)

Emission / deposition	Selin et al. 2007	Mason, 2008	Cordy et al. 2011	Mason et al. 2012
Natural from the land	0.5	–	0.3	0.08–0.6
Re-emission of the land	1.5	–	1.37	1.7–2.8
Biomass burning	–	–	–	0.3–0.6
(A) Together with the land	2.0	1.85	–	–
Natural from the oceans	0.4	–	–	–
Re-emission of the oceans	2.4		3.2	2.0–2.9
(B) Along with oceans	2.8	2.6	–	–
(C) Primary anthropogenic	2.2	–	1.9	2.0
All (A + B + C)	7.0	–	6.7	6.1–8.9
(D) Deposition on the lands	–	–	–	3.2
(E) Deposition on the assessment	–	–	–	3.7
All (D + E)	7.0	6.4	–	6.9

### Natural Emission Sources

Mercury emissions from natural sources into the atmospheric air are an important element of its global fluxes and the largest element of the global mercury cycle. Many research teams take efforts to estimate these emissions, using, apart from direct measurements, different numerical models. In general, natural emissions can be divided into primary emissions, e.g. volcanic emissions, and secondary emissions of natural origin. The balance is distorted by the share of re-emissions from anthropogenic sources which is difficult to estimate. Table 3 shows natural sources and re-emission processes which release mercury into the atmosphere, including volcanic activity, soils, surface waters, processes unfolding in the Earth’s crust and fires (Mason et al. 2012).

**Table 3:** Mercury emission from natural sources and processes estimated for 2008 (Pirrone et al. 2010a, b)

Source	Annual emission Mg/year	Relative contribution (%)
Oceans	2682	50
Lakes	96	2
Forests	342	6
Tundra/grassland/savannah/prairies/chaparral	448	8
Desert/metalliferous/non vegetation zones	546	10
Contaminated sites (average between 138 and 263 Mg	200	4
Agricultural areas	128	2
Evasion after mercury depletion events	200	4
Biomass burning	675	12
Volcanoes and geothermal areas	90	2
All	5207	100

In the oceans, mercury mainly occurs in the forms of Hg^0^, Hg^II^ and methyl forms (CH_3_Hg^+^ and CH_3_)_2_Hg) (Morel et al. 1998). The mercury emission levels from surface waters (Mason and Sheu 2002; Pirrone et al. 2003) mainly depend on the forms of dissolved mercury, particularly Hg^0^, the intensity of solar radiation and water temperature. They also vary, depending on the time of the day, and they are higher in daytime than in night-time. As a rule, the emissions from lakes are more intense. This is caused by higher concentrations of soluble forms of organic carbon in lakes (Boudala et al. 2000). Based on their research in lakes in the Canadian Province of Ontario, Lalonde et al. (2002) estimated that 40–54% of mercury was re-emitted within 24 h of its deposition. The process of photoreduction of mercury to the species Hg^0^ primarily involves solar radiation in the UVB range, while the share of waves in the other ranges is negligible (Lalonde et al. 2002, 2003).

In snow, mercury mainly occurs in oxidized forms: Hg^II^, HgC_2_O_4_, Hg(OH)_2_ and HgOHCl, while less than 1% of elemental Hg^0^ can be found in it (Poulain et al. 2004). Ferrari et al. (2008) estimated that in the Arctic conditions 50–80% of mercury is re-emitted into the atmosphere within 1 day.

Emissions from soils have the form of GEM and depend on many factors (Carpi and Lindberg 1997, 1998; Zhang et al. 2001; Ferrari et al. 2002; Gustin et al. 2002; Poissant et al. 2004):

- The properties of soils: mercury content, the contents of organic compounds
- The concentrations of oxidants, mainly ozone, in the air
- The weather conditions: solar radiation, temperature and winds

It is most difficult to estimate mercury emissions from plants which mainly occur in the form of Hg^0^ (Gustin 2003; Gustin and Lindberg 2005; Stamenkovic and Gustin 2007). The highest emissions come from tropical forests and savannahs (Mason 2009). Peat bogs are characterised by a relatively high mercury content, and therefore, their fires can, in particular, cause its higher emissions (Turetsky et al. 2006). The plant cover of soil, e.g. grass, can reduce emissions from the areas of substantially contaminated cities even to 26% (Coolbaugh et al. 2002).

Nriagu and Becker (2003) collected data from 200 publications and 10,000 single measurements of mercury emissions from 70 active volcanoes and 45 ones where only gas exhalation occurred, in the period from 1980 to 2000. The highest emissions came from the volcanoes: Bagana on Bougainville Island in the archipelago of Solomon Islands—99.2 t, Kilauea in Hawaii—86.7 t and Rabaul on New Britain Island in Papua New Guinea—80.7 t. Schuster et al. (2002) determined the mercury content in ice cores in the State of Wyoming (USA). Its levels were clearly correlated with the eruptions of large volcanoes.

Mason (2009) and Mason et al. (2010) presented their generalised findings on mercury emissions from natural sources:

- Sixty percent of mercury emissions from surface waters come from the Atlantic, Indian and Pacific Oceans.
- The largest mercury amounts are emitted from tropical and sub-tropical regions—45%, followed by those from the moderate zones—41% and the lowest emissions come from the polar regions—8%. The emissions from volcanoes and geothermal areas represent 2%.
- Based on emission levels, the areas can be arranged in the following order: deserts > forests > other areas (tundra, savannahs, chaparral) > farmland.

### Emissions from Anthropogenic Sources

Since the times before the scientific and technological revolution, mercury emissions have grown about three times and in highly industrialised regions, up to 10 times (Hylander and Meili 2003). In their report to the US Congress, Keating et al. (1997) estimated that in 1995, the emissions from anthropogenic sources represented 50 to 75% of the total emissions which amounted to 5500 t. At the end of the 1990s, the interest in the contamination of the environment by mercury greatly grew, contributing to more detailed research on its emissions and actions to reduce them.

The anthropogenic emission levels were determined on the basis of inventories of the mercury emissions and consumption in the particular sectors of the economy and states. The calculations were also assisted by increasingly advanced models. Still, the imperfection of the methods applied caused very large differences among the data given by different authors (Pacyna et al. 2008). Kindbom and Munthe (2007) developed a detailed methodology which is now often used. In addition to the emissions from industrial processes, it also took into account those from the following:

- The use of mercury-containing products
- Waste management (incineration, storage at landfills recycling);
- Residues in products used by man

Emission factors specific of production process types and mercury contents in raw materials are helpful in estimating its emissions (Pacyna et al. 2006, 2008, 2009, 2010a, b; Streets et al. 2009; Pirrone et al. 2010a, b). They are expressed by the ratio between the level of emissions into the atmosphere and the mercury content in a raw material or product. The highest values of these factors are characteristic of the production of non-ferrous metals: zinc (7.8–8.0), copper (5.0–6.0) and lead (3.0) as well as sewage sludge incineration (5.0). Coal combustion in public supply power plants is characterised by a relatively low factor (0.04–0.3), which is lower than that of coal combustion in the municipal and housing economy (0.1–0.5), but given the huge amounts of coal burned, its share in emissions determines to a large extent the contamination of the air by mercury.

The levels of mercury emissions into the air are significantly affected by its emissions from human activities directly using mercury and its compounds as a raw material or catalyst. In this context, the following processes can be listed (Pacyna et al. 2008):

- Gold mining and processing
- Catalysis in the production of plastics
- Chlor-alkali production
- The production of batteries
- Dentistry
- Control and measurement equipment
- Lamps
- Electrical and electronic devices
- Others (the production of pesticides and fungicides, preservatives in paints, chemical reagents, catalysts, cosmetics, applications in traditional medicine and those related to cultural activities and ritual ceremonies)

The second category of mercury contamination sources of anthropogenic origin includes the processes where mercury is an impurity in raw materials (Pacyna et al. 2008):

- Energy generation processes
- Cement production
- Waste incineration
- Steel and non-ferrous metals production
- Cremation

Table 4 shows total mercury emissions into the air, broken down into different human activities. The artisanal and small-scale gold mining (ASGM) has the largest share of 32.0%. At least 100 million persons in more than 55 countries, mainly in Asia, South America and Africa, rely on ASGM for their livelihoods (Telmer and Veiga 2009). The emissions originating from fuel combustion for energy generation purposes represent 28.3% (including 27.9% from coal combustion). The share of petroleum derivatives is slight, amounting to 0.4% only, while natural gas combustion is not taken into account. Metallurgy emits 13.2% of mercury, including the production of zinc—8.1%, lead and steel—2.2%, as well as the others grouped as aluminium, lead and copper—1.9%. The share of the cement industry, estimated at 10.8%, is also important. Taken together, these four human activities emit 84.3% of mercury (Wilson et al. 2012). According to other estimates, in 2010 (Zhang et al. 2016) the global GEM emissions represented 65% of emissions, including energy generation—47%, industrial processes—27% and ASGM—26%. The share of GOM in the global emissions was 35%. In 2014, in the Member States of the European Union (European Union 2016), 73% of emissions originated from energy generation, 20% from industrial processes and 5% from waste landfills.

**Table 4:** Global emissions of mercury to the atmosphere from anthropogenic sources divided into different areas of human activity (Wilson et al. 2012)

Sector	Emissioma (Mg/kg)		
	Average	Range	%
Coal combustion—together	573.9	116.1–820.7	27.9
Power	415.7	267.4–594.5	20.2
Industry	102.2	64.7–146.2	5.0
Municipal	56.0	35.4	2.7
Burning petroleum derivatives—together	9.3	4.3–15.3	0.4
Power	3.7	1.7–6.1	0.2
Industry	3.0	1.4–5.0	0.1
Municipal	2.6	1.2–4.2	0.1
Metallurgy—together	–	–	–
Including iron and steel	45.4+	16.0–88.4	2.2
Aluminium	5.9	2.1–11.6	0.3
Copper	20.3	7.2–39.2	1.0
Lead	32.4	11.6–62.7	1.6
Zinc	166.9	59.5–322.9	8.1
Mercury production	9.0	3.2–17.6	0.4
Cement	223.1	79.5–431.6	10.8
Production of caustic soda and chlorine	52.0	18.5–100.8	2.5
Refineries	49.9	23.1–82.4	2.4
Gold production on a large scale	93.7	0.7–245.9	4.7
Artisnal and small-scale gold (ASGM)	659.4	409.7–906.2	32.0
Incineration of waste—organised	4.2	1.3–12.7	0.2
Cremation	4.8	1.4–14,3	0.2
Other	109.1	32,7–327,2	5.3
All	2063	1038–3499	100

Table 5 (Wilson et al. 2012) shows total mercury emissions from anthropogenic sources, broken down into continents and regions. The countries of East and South-East Asia had the overwhelmingly largest share, amounting to as much as 45.7%, followed by South Africa—10.9%, South America —10.4% and South Asia—9.5%. These high shares of Asian countries and South America are related to a large scale of ASGM located there. Zhang et al. (2016) demonstrated large differences among continents: in Western Europe, GEM represented 80% and GOM 20%; in North America, GEM 88% and GOM 12%; whereas in Asia and Oceania, GEM 88% and GOM 37%.

**Table 5:** Mercury emissions from anthropogenic sources in 2012 of the continents and regions (Wilson et al. 2012)

Continents—regions	Emission (t)	%
Australia, New Zealand, Oceania	25.4 (5.6–56.6)	1.2
Central America and the Caribbean	42.8 (21.1–73.7)	2.1
Middle East USA	45.2 (20.4–85.3)	2.2
Americas	90.0 (48.4–156.3)	4.4
South America	215.5 (101.4–335.1)	10.4
CIS and other European countries	123.3 (51.8–233.7)	6.0
European Union	141.6 (68.2–253.4)	6.9
East and South-East Asia	942.4 (478.5–1583.1)	45.7
South Asia	195.9 (106.0–326.5)	9.5
North Africa	15.5 (20.4–85.3)	0.7
Sub Saharan Africa	225.8 (131.5–364.0)	10.9

#### Gold and Silver Mining and Processing

The contamination of the environment by mercury related to the technology used to produce pure forms of gold and silver was already known in the ancient times. This process is relatively simple and cheap, consisting in leaching and amalgamation of the metals from ores. The use of mercury in this process of obtaining gold and silver consists in dissolution of the metals in mercury where they form amalgams, from which they are then recovered through its evaporation (Lacerda 1997). It was probably the Romans who used the process for the first time in about 50 A.D., and the annual mercury consumption is estimated at more than t/year (Nriagu 1996).

In modern times, the Spaniards were the first to use the amalgamation technology in Mexico (from 1554) and then in Peru and Bolivia, mainly to produce gold (Nriagu 1993). Lacerda (1997) estimated the emission factor of 1 kg of Au/1 kg of Hg and van Straaten (2000) 1 kg of Ag/1.2–1.5 kg of Hg. Nriagu (1993, 1994) estimated the total mercury emissions into the environment in Latin America in 1554–1880 from the production of gold and silver at 196,000 t and the annual emissions at 612 t/year (292–1880 t). He estimated its emissions into the air in 1580–1900 in North, South and Central Americas and Australia at 156,000 t and those into the environment at 250,000 t. More recent research suggested that the emissions estimated earlier for the “gold rush” period had been overestimated even by as much as 50% (Strode et al. 2007).

In the early 19th century, in North America and Australia gold and silver began to be mined on a large scale, in a process called a “gold rush”, which came to an end in the early 20th century. The mining areas in the United States were situated in California, Nevada, North Dakota, the Carson River Valley and, to a lesser extent, in the mountains – the South Mountains in North Carolina, in Nova Scotia in Canada and in the Bandigo goldfields in the state of Victoria in Australia. The total mercury emissions in the USA caused by the “gold rush” in 1840–1900 was estimated at 60,000 t, i.e. about 1000 t/year (Bloom and Porcella 1994: Nriagu 1994).

In the 20th century, the amalgamation technology in the industrial gold production was gradually replaced by a cheaper method using cyanides. This new technology did not require the use of mercury in the production process. The amalgamation technology ceased to be applied on a large scale in Canada and Wales (Great Britain) in 1916, in Australia in 1930 and in the United States in 1950 (USGS 1968; Fuge et al., 1992).

Following a surge in gold prices on the world market from USD 58 for an ounce in 1972 to USD 447 for an ounce in 1987, a new “gold rush” began in developing countries. In this case, gold is produced mainly by the amalgamation method in small and very small production plants (Lacerda and Salomons 1991; Lacerda, et al. 1991; Roulet et al. 1999).

In the literature, this source of mercury emissions is called “Artisanal and Small-Scale Gold Mining” (ASGM). It is a sector of the economy which consumes the largest amounts of metallic mercury, i.e. about 1.000 tonnes annually. This represents more than 30% of mercury consumption in all the industrial processes (Swain et al. 2007).

The annual mercury emissions from this source into the environment are estimated at 650–1350 t/year, on average 1000 t/year. Within them, the direct emissions into the atmosphere are estimated at 650 t/year, while the remainder ends up in rivers, lakes, soils and waste landfills. In the world, there are 10–15 million small companies in 70 countries which mine and process gold. Annually, they produce more than 350 tonnes of this precious metal (Telmer and Veiga 2009). The emissions consist of GEM—80.0%, GOM—14.9% and TPM—5.1% (Feng 2005).

It is difficult to estimate the amounts of mercury which end up in the atmosphere as a result of re-emission. China, Brazil, Indonesia, Columbia, Bolivia, Venezuela and Philippines emit much larger amounts of mercury from these sources, while the emissions from other countries are much less significant. More than half the mercury used for these purposes is consumed in South-East and South Asia and one fourth of it in South America (Pacyna et al. 2008).

In Columbia, 200.000 persons are employed in gold mining and processing, to produce 30 tonnes of gold annually, according to official data. In its region of Antioquia, there are 17 mining centres which employ up to 30.000 persons and directly in the course of the production process about 50% of mercury is emitted into the atmosphere. The mercury concentrations in cities in this region reach extremely high levels: the background level of 300, 1.0000 ng/m^3^ in housing districts and up to 1 million ng/m^3^ in gold processing facilities (Cordy et al. 2011).

Before 1915 the amalgamation technology for gold and silver production had been the overwhelmingly largest source of mercury emissions. After 1950 its share has decreased but still continues to be significant (Streets et al. 2011).

#### Chlor-alkali Industry

The chlor-alkali industry produces chlorine (Cl_2_) and alkali: sodium hydroxide (NaOH) or potassium hydroxide (KOH), through the electrolysis of solutions of salts (chlorides of akali metals). The basic technologies applied in chlor-alkali production include electrolysis in the mercury cell (the mercury process), the diaphragm cell (the diaphragm process) or the membrane cell (the membrane process), mainly using sodium chloride (NaCl) and potassium chloride (KCl) as a raw material. This industry produces chlorine, caustic soda (NaOH) and, to a lesser extent, potassium hydroxide (KOH). In 1887, the global chlorine production reached 115 tonnes (with chlorine mostly used for bleaching), while at present it amounts to almost 50 million tonnes and continues to grow (Lindberg and Turner 1977; Kinsey et al. 2004).

In Europe, 50 installations producing chlorine using mercury were in operation in 2005, with their largest number in Germany, i.e. 10, with 9 installations in France and just as many in Spain (Pirrone et al. 2009). The production technology is applied in two stages. The first stage involves the electrolysis of saturated brine (NaCl) with the participation of mercury as an electrode. The liberated chorine is stored, while metallic sodium forms an amalgam with mercury. The second stage entails the decomposition of the amalgam into metallic sodium, which reacts with water to form sodium hydroxide (caustic soda), hydrogen and metallic mercury. The electrolysis operation follows the reaction (Kinsey et al. 2004). In 1887, the global chlorine production reached 115 tonnes (with chlorine mostly used for bleaching) and in 2010 it amounted to 50 million tonnes. In 2010, the global mercury emissions related to the chlor-alkali industry were 52.0 t/year (18.5–100.8 t/year), representing 2.7% of its total emissions from anthropogenic sources (Lindberg and Turner 1977; Kinsey et al. 2004; Wilson et al. 2012). Based on the earlier data from 2000, the emissions were estimated at 65.1 t/year (Pacyna et al. 2006; Feng et al. 2009; Mukherjee et al. 2009).

The production of a tonne of chlorine consumes about 200 g of mercury (Mukherjee et al. 2009). In Europe, it is estimated that the production of a tonne of chlorine causes the release of 0.2–3.0 g of mercury into the atmosphere (Pirrone et al. 2009. These emissions consist of GEM—70%, GOM—30% and TPM ∼ 0% (Pacyna and Pacyna 2002).

In many countries, mercury emissions into the air can be seen to diminish as a result of a change of the production methods consisting in the replacement of the mercury-based method by the membrane-based one. In India, at the end of the 20th century the mercury-based production of Cl and NaOH dominated, but in 2004 it was applied at only 16% of installations and in 2012 the mercury-based method was abandoned (Mukherjee et al. 2009). In the USA, the level of emissions in 1990 was 10.0 t/year, whereas in 2002 it was already only 5.4 t/year (Pirrone et al. 2009); in turn, in India in 2000 it was 132 t/year, whereas in 2004 it was only 6.2 t/year (Mukherjee et al. 2009.

The factories producing Cl and NaOH also contribute to substantial contamination of not only the air, but also soils, plants and surface waters, e.g. in the vicinity of a factory in the Portuguese town of Estarreja, which had then produced for more than 50 years, the mercury content in soil reached the level of as much as 91 mg/kg and that of 2 mg/kg in *Lolium perrene*. However, its contamination level of importance for human health occurred only in the area directly adjacent to the factory (Reis et al. 2009).

#### Electrical and Electronic Devices

Mercury and its compounds are used in the production of electrical and electronic devices. They include, in particular, switches, relays and other devices of this type. In 2005, the global mercury consumption in this sector of the economy was estimated at 200 t/year (180–220 t/year). In recent years, this level distinctly decreased (Pacyna et al. 2008). Directive 2002/95/EC of 27 January 2003 on the restriction of the use of certain hazardous substances in electrical and electronic equipment (the Restriction of Hazardous Substances – RoHS – Directive) was in effect in the Member States of the European Union from 1 July 2006 and was replaced by RoHS 2011/65/EU, which entered into force on 3 January 2013. The purpose of these legal acts is to reduce the amounts of hazardous substances penetrating into the environment from landfills of waste electrical and electronic equipment. The scope of application of the Directive includes restrictions on the introduction of hazardous substances in electronic devices at the production stage and provides for the sound collection and disposal of waste electrical and electronic equipment. Thus, it covers the whole “lifecycle” of the products to which it applies. Similar legal acts are in effect in China, Japan and the USA (Pacyna et al. 2008).

#### Lighting Equipment

Some lighting equipment contains mercury. It consists of different types of lamps: fluorescent (glow, neon), UV, CFL, high-intensity discharge, sodium and other lamps. Fluorescent lamps contain the largest amount of mercury, i.e. up to 2.500 mg/lamp. More recent lamps contain much less mercury. A serious problem arises when their use ceases and they are deposited at waste landfills (Maag et al. 1996, 1997). Their correct disposal is difficult because of the dispersal of their users. e.g. in Japan only 7.2% was recycled (Asari et al. 2008).

Wilson et al. (2012) estimated the global mercury consumption in 2010 in the production of lighting equipment at 123 t (105–135 t). The largest consumers included the countries of South-East and East Asia—42 t (38–45 t), the Member States of the European Union – 17 t (13–20 t) and the countries of North America—15 t (12–18). In 2005, Pacyna et al. (2008) estimated this global consumption at a higher level, i.e. 120 t (134–150 t).

The threats posed by the environmental contamination by lighting equipment deposited at waste landfills induced many countries to adopt restrictive legal regulations to limit the use of mercury in the production of lamps. In the USA, the actions by the Environmental Protection Agency (EPA) caused the share of mercury from lamps at waste landfills to decrease to 3.8%. On the other hand, in the course of waste incineration more than 90% of mercury was released into the air (EPA 1992). In the period from 1999 to 2005, the recycling level grew there from 2 to 25%, while the annual emissions fell from 5.5 t in 1990 to 1.0 t in 2005 (Cain et al. 2008). In Europe, 4 t of mercury from lamps was deposited annually at waste landfills (Mukherjee et al. 2004).

#### Batteries

Many battery types contain mercury. The global annual mercury consumption in 2010 in the production of batteries was 291 t (230–350 t), while the largest consumers included the countries of South-East and East Asia—191 t (153–228 t), South Asia—26 t (17–34 t) and the Member States of the European Union—20 t (17–27 t) (Wilson et al. 2012).

The restrictive regulations in effect in many countries substantially reduced the mercury consumption for battery production. In 1990–2005, the global annual consumption for this purpose decreased from 1.720 to 365 t (Wilson et al. 2010). E.g. in the USA the annual emissions from this source fell from 101.2 t in 1990 to 0.5 t in 2005 (Cain et al. 2008). In China, this reduction was also significant—from 47.9 t in 1997 to 3.7 t in 2003 (Feng et al. 2009).

#### Coal

Depending on its type and origin, coal mainly contains mercury in the form of inorganic compounds: sulphides (HgS, cinnabar), chlorides and sulphates. Its remainder occurs in organic compounds. However, the proportions between inorganic and organic compounds are very different (Groen and Craig 1994; Finkelman 1994; Galbreath and Zygarlicke 2000).

In Poland, the largest amount of mercury was contained in hard coal from the Nowa Ruda Mine in Lower Silesia, depending on the deposit, from 0.81 to 9.67 mg/kg, on average 3.99 mg/kg (Bojakowska and Sokołowska 2001). Lignite from Polish deposits contains four times more mercury than hard coal does (Pasieczna et al. 2007). Analysing data on the mercury contamination of hard coal, Yudovich and Ketris (2005) determined that the highest mercury contents could be found in the mines in the Donetsk Coal Basin in Ukraine, the deposits in the Chinese Province of Guizhou and the American ones in the Appalachians, in the coal called Upper Freeport in the USA, in its deposits situated close to the ground surface (Richaud et al. 1998).

Compared with other countries, the hard coal in Australian deposits contains extremely low amounts of mercury, on average 0.04 mg/kg (0.01–0.13 mg/kg). A low sulphur content in coal was suggested as the cause of this (Nelson 2007). Similarly, the deposits in the Prince Charles Mountains in eastern Antarctica contain on average 0.04 (<0.02–0.14 mg/kg) (Chiehowsky et al. 2003). To a large extent, mercury in coal can be found in its compounds with sulphur; therefore, the content of the latter determines that of mercury (Swaine 1994). The research by Quick et al. (2003) and Zhang et al. (2009) demonstrated a strict correlation between the contents of mercury and sulphur in coal.

In the waste gases from coal combustion, 99% of mercury occurs as GEM (Furimsky 2000). After the waste gases are cooled to less than 400 °C, this mercury species can react with other compounds, to partially transform into GOM (Sondreal et al. 2004). In the Chinese Province of Guizhou, there is the following mercury speciation in waste gases: GOM—46%, GEM—31% and TPM—8% (Tang et al. 2007).

In the USA, in the waste gases from coal-fired power plants, on average mercury is present as: GEM—54%, GOM—43% and TPM—3% (Leopold 2002). The volatile particulate matter generated by coal combustion is characterised by its very high capacity to absorb different mercury species, which is even higher than activated carbon (Otani et al. 1986; Schager et al. 1992; Hall 1995).

In waste gases, a number of reactions unfold to determine mercury species. The course of a reaction depends on the temperature as well as other compounds and elements present in the waste gases. In these processes, the share of chlorine compounds is decisive. The content of this element in coal varies in a wide range, depending on the deposit and coal type. For example, in the USA, the chlorine content in bituminous coal can reach even 1000 mg/kg, whereas it is much lower in sub-bituminous coal and lignite, i.e. 100–200 mg/kg (Sondreal et al. 2004). In hard coal from six Chinese mines, its content varied between 152 and 875 mg/kg (Lei et al. 2007).

The global mercury emissions into the atmosphere from coal combustion in 2010 amounted to 574 t/year (116–821 t/year), representing 27.9% of the total emissions from its anthropogenic sources. Power plants had the largest share of 20.2%, the other industrial sectors had a smaller share of 5.0%, while the share of household combustion, 2.7%, was the smallest (Wilson et al. 2012).

#### Crude Oil

Crude oil contains much less mercury than coal does. Its content varies depending on the place of origin and may be different between deposits even by several orders of magnitude. The large variability even within one deposit is evidenced by the mercury content in the Cymric Oil Field in California, varying between 1 and 1.560 ng/g (Magaw et al. 1999) or in the deposit in the Canadian Province of Ontario, varying between 0.1 and 44 ng/g (Hollebone and Yang 2007). According to the majority of authors, the mercury content falls within the interval from 1 to 5 ng/kg. However, the earlier research done in the 1970s indicated much higher values (Shah et al. 1970; Heinrichs 1975).

The mercury content in refined products substantially varies even within one fraction. Its content in heavy petrols is the largest, up to 40 ng/g (Olsen et al. 1997), and in coke, up to 50 ng/g (Wilhelm 2001). According to Hoyer et al. (2004), petrol contains less mercury than diesel oil, respectively, 2.5–11.4 and 70.9–123.8 ng/L. Landis et al. (2007) gave a contrary proportion: petrol—62 ng/L and diesel oil—284 ng/L. Mercury in the GEM species dominates in crude oil. According to Bloom (2000), there are the following proportions between the different mercury species: GEM—76%, GOM—33% and MeHg— <1%. The mercury emissions into the environment from the crude oil refining process consist of: its direct emissions into the GOM—33% i MeHg— <1%.

The issue of mercury in the environment in the process of refining crude oil consists of direct emission to air, waters, solid waste and the mercury contained in products. In the USA, 20% of mercury is emitted into the air, 25% into waters and in the form of solid waste, while 55% remains in products (Wilhelm 2001).

Pirrone et al. (2009) estimated the global mercury emissions from petrol and diesel oil combustion at 378 kg/year (192–564 kg/year), including 238 kg/year for petrol (121–281 kg/year) and 140 kg/year (71–209 kg/year) for diesel oil. The highest emissions took place in North America, i.e. 155 kg/year. These authors estimated the share of petrol combustion in the global emissions into the air from anthropogenic sources at only 0.0015%, pointing out that the calculations might be very inaccurate, given the low reliability of data from many countries. In the USA, in 2002, in its National Emissions Inventory, the Federal Environmental Protection Agency (EPA) estimated the share of motor vehicles in the annual national emissions only at less than 1%, i.e. 1 t/year (Pirrone et al. 2009). The estimated mercury emissions from the combustion of crude oil used to propel vehicles did not take into account the contributions from aircraft, ships, military vehicles and biodiesel combustion (Streets et al. 2011).

#### Natural Gas

Just as crude oil, natural gas contains slight amounts of mercury. It occurs almost completely as GEM, whereas organic mercury compounds have a substantial share in condensates and petroleum derivative liquids and can even be a dominant species of this element (Edmonds et al. 1996; Zettlizer et al. 1997). The mercury contents in gas from deposits in different regions of the world are greatly varied: from 1 μg/m^3^ in the Norwegian deposits in the North Sea (Carnell et al. 2007) to even as much as 5000 μg/m^3^ in German deposits (Zettlizer et al. 1997).

The gas purification process mainly consists in removing CO_2_ and H_2_S, which is also accompanied by the removal of mercury. Thus, the gas delivered to users contains slight amounts of mercury and global inventories do not take into account the share of mercury emissions from gas combustion (Pacyna 1996; Pirrone et al. 2010a, b).

#### Cement Production

The industrial cement production sector is mentioned as a significant source of the environmental contamination by mercury. The production process consists in the burning of powdered and mixed raw materials (limestone, gypsum, shales, coal, sand). The high temperature of the process, reaching 1000 °C, is favourable for mercury emissions. The global mercury emissions from cement production are estimated on average at 223.1 t/year, in a relatively wide range from 79.5 to 431.6 t/year, representing about 10.8% of the total emissions of anthropogenic origin (Wilson et al. 2010). In 2005, Asia excluding Russia had the overwhelmingly largest share, i.e. on average 72%, and was followed, in turn, by Europe excluding Russia, with its share of 9.9% (Pacyna et al. 2010a, b). Among states, China has the largest share, of 14.8%, in the global emissions from this sector of industry and 5.7% of the total emissions from this country (Zhang and Wong 2007; Feng et al. 2009; Streets et al. 2009). At the same time, the cement production in China can be seen to grow very quickly. Wu et al. (2010) determined the rate of this growth in the period from 2004 to 2007 as 5.9% annually.

The mercury emissions from cement production consist of: GEM—80%, GOM—15% and TPM—5% (Streets et al. 2005; Pacyna et al. 2008). According to the calculations by Pacyna et al. (2006), the emission factor is 1 g of Hg per the production of 1 t of cement.

#### Cremation

The cremation of bodies is also a source of mercury emissions into the air. This is related to the use of amalgams in dentistry to fill in cavities in teeth. According to Wilson et al. (2012), in 2010 its share of the global emissions was barely 0.2%, i.e. in absolute values on average 4.8 t/year (1.4–14.3 t/year), and, according to Pacyna et al. (2010a, b), in 2005, it was 26 t/year. In the world, the practice of cremation is related to customs and beliefs, e.g. it is very seldom used in Muslim and Greek Orthodox countries. In Japan, 99.9% of bodies are cremated (Takaoka et al. 2010) and the mercury emissions into the air from persons who died at the age of 66–65 years amount on average to 161 mg/body, while those at the age of 0–59 lat emit less than 20 mg/body (Takaoka et al. 2010). In Europe, single bodies contain 2–5 g of mercury (Pacyna et al. 2008). The largest contributors to the emissions from this source are the countries of East and South-East Asia, with 16 t/year, and Europe, with 3.75 t/year (Cain et al. 2008; Pacyna et al. 2010a, b). The waste gases from crematoriums contain on average mercury in the form of: GEM—80%, GOM—15% and TPM—5% (Pacyna et al. 2008).

#### Iron and Steel Production

In iron and steel production, mercury emissions are related to high-temperature manufacturing processes using numerous raw materials and the emission levels depend on the mercury contents in them. Ironworks and steelworks emit mercury in gaseous form (GEM—80% and GOM—15%) and particle-bound mercury TPM—5% (Wilson et al. 2012). The global annual emissions from this source in 2010 were estimated at 45.4 t, representing 2.2% of the total emissions from anthropogenic sources (Wilson et al. 2012). Compared with 2005 there was a decrease by 22.4% (Pacyna et al. 2008). According to other estimates (Wilson et al. 2012), in those years the emissions grew by 20.4%, with the greatest increase in South and South-East Asia by 51.3% and a decrease in North America by 25% and in the Member States of the European Union by 13.2%.

#### Non-ferrous Metal Production

Ores of non-ferrous metals: zinc, lead, copper and gold, contain admixtures of other metals recovered in the production process (silver, nickel, gold etc.). Mercury is also an admixture which commonly occurs in ores, but which, however, is undesirable. Its content varies greatly in ores depending on their type. This variation is related to different generations of ore mineralisations occurring in these deposits (Mayer and Sass-Gustkiewicz 1998). According to Streets et al. (2005), Asian zinc ores contain 86.6 t/g, whereas according to Pacyna and Pacyna (2002), this content is 20.0 g/t. Gold ores from RSA contain a good deal more mercury, i.e. as much as 0.6–5.8% (Frimmel and Gartz 1997).

According to Theloke et al. (2008), zinc production is characterised by much higher mercury emissions per unit of metal produced than that of lead and copper. There is the following mercury speciation in the waste gases from smelters of non-ferrous metals: GEM—80%, GOM—15% and TPM—5% TPM (Pacyna and Pacyna 2002).

The following factors determine the emission levels (Pacyna et al. 2006):

- The mercury content in the ore
- The type of the primary technological process and possible use of scrap
- The type of emission abatement equipment

Pacyna et al. (2010a, b) estimated the global mercury emissions from the production of non-ferrous metals (zinc, copper and lead) in 2005 at 141 t, but according to Hylander and Herbert (2008) already at 275 t and Pirrone et al. (2010a, b) estimated at 275 t. China is characterised by far the largest and increasing share to 147.6 t in 1999 and 203.3 t in 2003 (Streets et al. 2005; Feng et al. 2009).

In the past, large amounts of mercury were emitted during its mining and processing. E.g. the mercury emissions from the processing plant in Huancavelica in Peru in the period from 1564 to 1810 amounted to about 17.000 t (Robins et al. 2012). At present, the emissions from the primary supply and processing of mercury are characterised by a relatively low emission factor of 0.2 with the speciation of GEM—80% and GOM—20%, with the global emissions in 2005 amounting to 8.8 t/year (Pacyna et al. 2008).

#### Waste Incineration

In 2010, the global annual emissions from (organised) waste incineration was estimated at 4.2 t (1.3–12.7 t), representing 0.2% of the emissions from anthropogenic sources (Wilson et al. 2012). The mercury emissions into the air from incineration plants depend on the composition of waste, the technologies applied and the equipment designed to reduce pollutants in waste gases. The incineration process is usually carried out at high temperatures of about 1000 °C and, as a result, almost all mercury is transformed into the gaseous phase. Municipal waste contains particularly much mercury, since it contains devices deposited at waste landfills, such as: thermometers, batteries, different types of lamps, mercury relays, waste from dental and medical clinics, measuring devices etc. Sewage sludge from wastewater treatment plants can be a significant source of mercury. The emission factors of the incineration of municipal waste and sewage sludge are relatively high, i.e. 1 and 5, respectively (Streets et al. 2009; Pirrone et al. 2010a, b). Restrictive legal regulations prohibiting their deposition at waste landfills and imposing the obligation to recycle mercury-containing objects contributed to reductions in the emissions from this source in developed countries. For example, in the USA, in 1995–1997, it proved possible to reduce the emissions from incineration plants burning municipal and medical waste by 90–95% (Stokstad 2004).

#### Other Sectors

This category includes the use of mercury in other sectors of the economy which have not been described earlier and those related to other human activities. In agriculture, mercury compounds are used in the production of fungicides and pesticides, and they are also applied as preservatives in the production of paints and catalysts in the production of plastics (other than vinyl chloride production) in the tanning industry and in the production of dyes and fireworks. Relatively, many mercury compounds are used as reagents in laboratories and in cosmetics to produce lightening creams. In Latin America and India, they are applied in cultural and religious ceremonies, while in China they are used in folk medicine (UNEP 2004; Pacyna et al. 2008).

It is practically impossible to accurately determine the levels of mercury consumption and emissions related to these sources. Pacyna et al. (2008) estimated the mercury consumption in these human activities at 313 t/year (225–400 t/year), with the largest share of 113 t/year contributed by the Member States of the European Union.

Mercury compounds are used as a catalyst in the production of vinyl chloride, a raw material applied in the manufacture of polyvinyl chloride (PVC), one of the basic plastics. In China, in 2004, the consumption of mercury for the purposes of this production amounted to 610 t/year and it is estimated that the demand for PVC has grown from year to year by 24–30% (Pacyna et al. 2008). One of the greatest ecotoxicological disasters was related to PVC production. It took place in the 1950s in the Japanese town of Minamata on Minamata Bay. It was caused by water contamination with mercury compounds from the plastics factory of Chisso Corporation which used mercury compounds as catalysts. Officially, 2265 cases of illness were registered, including 1784 fatalities, and about 10000 persons received compensations from Chisso Corporation (Allchin 1999).

The global mercury emissions into the air which are related to PVC production are estimated at 64.6 t (Pirrone et al. 2010a, b), including 14.7 t/year in Europe (Pacyna et al. 2008), 7.7 t/year in China (Feng et al. 2009) and 7.5 t/year in India (Mukherjee et al. 2009).

In 2005, the global mercury consumption in the production of measuring and control devices was 350 t/year (320–350 t/year), most of which, i.e. almost 80%, amounting to 270 t, was related to the production of thermometers and sphygmomanometers (devices for indirect measurements of arterial blood pressure). Eighty to ninety percent of these devices are produced in China and exported to the whole world (Pacyna et al. 2008).

In dentistry, metallic mercury is used to prepare amalgams of other metals (silver, copper, tin), which are then applied to fill in tooth cavities. As a result of the activity of one dentist, about 3.4 g/day of mercury ends up in the environment and the emissions from this activity are related to the following Paryag et al. (2010):

- The cremation of bodies (direct emissions into the air)
- The release of mercury from bodies buried in the ground (emissions to the soil and groundwater)
- The release of mercury from waste generated at dentists’ offices (emissions to the soil, groundwater and the air)

The global mercury emissions into the air from this source in 2005 were estimated at 321 t. The greatest contributors included the Member States of the European Union—95 t, South-East and South Asia—70 t and North America (excluding Mexico)—36 t (Wilson et al. 2010). In many countries (Sweden, Denmark, Norway, Finland, Japan and the USA), the use of amalgams in dentistry was reduced by banning it fully or applying other materials for dental restoration (Pacyna et al. 2008). However, the assessment of the level of mercury emissions from this source is very inaccurate, due to the difficulty with determining the lifecycle of the product and its unspecified emission factor (UNEP Chemical Branch 2008).

## Changes in Mercury Emissions in Contemporary Times

The estimation of global emissions of anthropogenic origin involves a very high level of uncertainty. Emission inventories are imperfect because of hardly precise estimation methods, the practical absence of estimation in many countries and failure to consider certain fields of activity (Pacyna et al. 2008). Streets et al. (2011) estimated the global anthropogenic mercury emissions into the air until 1850 at 137,000 t. The largest shares were contributed by the production of: silver—57.5%, mercury—30.4%, gold (ASGM)—6.0%, zinc, lead and copper—5.4% and coal combustion—0.6%. The share of the other human activities did not exceed 2%. In the period from 1850 to 2008, these emissions amounted to 215.000 t. The shares of particular activities changed. The largest shares still continued to be contributed by the production of silver—31.3% and mercury—24.8%, but coal combustion took the third place with its 15.8%. The share of non-ferrous metals grew to 6.2% and new emission sources appeared: the industrial-scale gold production—9.6%, caustic soda production—2.0, cement production—1.4%, waste incineration—2.5% and the combustion of petroleum derivatives—0.5%.

Wilson et al. (2010, 2012) estimated verified data on changes in global emissions in the period from 1990 to 2010. On the basis of the data presented, it is difficult to identify trends. The highest emissions occurred in 2010 amounting to 2.063 t, and in 1990, their level was 1.967 t, while the lowest emissions came in 1995, amounting to 1.824 t.

In the Mediterranean region, in the period from 1983 to 1998, the emissions grew by 39% (Pirrone et al. 2001). In China, in the period from 1995 to 2003, the emissions were also found to increase by 22% and, just as in the Mediterranean countries, their growth was caused by waste incineration (Feng et al. 2009). In contrast, in India (Mukherjee et al. 2009), in the period from 2000 to 2004, the emissions fell by 27%. This was an effect of a change of technology in the chlor-alkali industry and the replacement of the mercury method by the membrane method, which reduced the emissions from this sector of industry from 123 t in 2000 to 6.2 t in 2004. Similarly, in Finland (Mukherjee et al. 2000), in the period from 1990 to 1997, the emissions fell by 84%, as an effect of lower emissions from industrial processes.

Considering continents and regions, until the First World War, the largest contributors to the emissions included North America and Europe as well as Australia and Oceania, whereas after the Second World War, the emissions in Asia overwhelmingly dominated and new large sources emerged in the countries of the former USSR and Africa. The positions of Europe and America substantially diminished, while that of Australia and Oceania became highly insignificant (Streets et al. 2011).

Forecasts of changes in the levels of mercury emissions from anthropogenic sources must be based on the following premises:

- The determination of the rate of the further socio-economic growth
- The knowledge of the emissions in the base year
- The technical capacity to reduce emissions from particular sectors
- A predicted change in the demand for given products
- Legal regulations limiting emissions
- The development of methods for controlling emissions
- Improved emission inventory methods
- Predicted changes in particular countries

Pacyna et al. (2008, 2010) and Pirrone and Mason (2008) presented forecasts of changes in the global levels of emissions from particular sectors until 2020, based on three scenarios, with 2005 used at the base year:

1. “Status Quo” (SQ)—a pessimistic scenario, providing for further socio-economic growth and higher emission levels in certain sectors.
2. “Extended Emission Control” (EXEC)—a scenario providing for further socio-economic growth, with simultaneous changes of many industrial technologies, and the global expansion of control methods and legal regulations applied in Europe and North America. The implementation of the recommendations of the Kyoto Protocol for combating climate change will also cause reductions in mercury emissions.
3. “Maximum Feasible Technological Reduction” (MFTR)—an optimistic scenario, providing for the implementation of all feasible methods for reducing mercury emissions.

Relative to 2005, in the pessimistic SQ scenario, the total emissions from anthropogenic sources in 2020 will grow by 25%. This will be caused by higher emissions in the sectors of fuel combustion and cement production. The levels of emissions from the other sectors will not change. The optimistic MFTR scenario provides for emission reductions by 45% compared with 2005, mainly in the sectors of waste incineration by 85%, iron and steel and non-ferrous metals production by 73% and cement production by 68%. All the three scenarios provide that until 2020, the chlor-alkali industry will not use the mercury-based technology, and therefore, this sector will cease to contaminate the environment with mercury. Given the dispersed, often artisanal, small-scale gold production (ASGM), the EXEC and MFTR scenarios do not provide for changes in the levels of emissions from this sector.

The mercury emission forecasts for 2050 by Streets et al. (2009) are much more pessimistic that those until 2020. They provide for emissions increasing by −4% do +96%, depending on the scenario applied. All the scenarios provide that the share of Asia will exceed 50% of the total emissions.

## Air Contamination by Mercury

Several standardised methods can be used for measuring mercury in the air, expecially from industrial emission sources or process streams. For example, atomic absorption spectroscopy (AAS), atomic fluorescence spectrometry (AFS), UV differential optical absorption spectroscopy (DOAS), inductively coupled plasma atomic emission spectrometry (ICP-AES) and inductively coupled plasma mass spectrometry (ICP-MS) techniques (Clevenger et al. 1997). Also, the CVAFS (atomic fluorescence spectrometry method using cold vapour) and CVAAS (atomic absorption spectrometry with cold vapor) based on gold trap amalgamation can be used for mercury detection (Zielonka et al. 2005). However, only two methods (AAS and AFS) are approved by the US-EPA (Das et al. 2001).

The transformations of the different mercury species occurring in the air were addressed in detail in **Chapter 1**. Gaseous mercury species represent about 98% of its mass in the air. It occurs in three oxidation states: Hg^0^, Hg^+^ and Hg^+2^. Elemental mercury (GEM) dominates, representing about 95% of its total mass; the second oxidation state can be found in small quantities, while the first oxidation state occurs in trace amounts (Schroeder et al. 1998). Oxidized mercury species can be found in the air at very low concentrations (pg/m^3^) and because of low vapour pressures they quickly undergo dry deposition to the surface (Schroeder et al. 1998; Mason 2005; Mason et al. 2010).

Mercury in organic compounds (Me_2_Hg) occurs mainly as MeHg and (CH_3_)Hg and (CH_3_)_2_Hg, with their shares in the contamination representing only 0.3–1% of the total mercury content in the air. E.g. in Gothenburg (Sweden) the MeHg concentrations from April to August 2000 amounted to 2–22 pg/m^3^, on average 7.4 pg/m^3^ (Lee et al. 2003). Ericksen and Gustin (2004) suggested the following hierarchy of environmental parameters influencing flux: soil moisture > light > air concentration > relative humidity > temperature.

Apart from its natural and anthropogenic emissions and re-emissions, the air contamination by mercury is driven by the rate of its dry and wet deposition. The deposition rate is affected by the weather conditions: the wind speed, air humidity, insolation, atmospheric precipitation, type of surface, concentration, mercury species and the forms of other pollutants in the air (Gustin 2012).

In the case of GEM, the residence time is estimated at 6 to 18 months, while GOM and TPM contained in particulate matter are quickly removed from the air through wet and dry deposition and their residence times are estimated at most at hours and days.

GEM and MeHg are incomparably less soluble in water than GOM is. This is related to the mercury residence time in the air. This time depends on many factors. Apart from the weather conditions and its different species, the air pollution is also important. In the case of GEM, the residence time is estimated at 6 to 18 months, while GOM and TPM are quickly removed from the air through wet and dry deposition and their residence times are estimated at most at hours and days (Selin et al. 2007; Skov et al. 2008; Mason et al. 2010). Given the long time of its removal from the air, GEM can be transported over large distances (Travnikov et al. 2008).

The particular mercury species are characterised by different dry deposition rates which also determine their residence times. The dry residence times of the different mercury species form the following series (Lindberg et al. 2002):

$$ \mathrm{GEM}\left(0.19\ \mathrm{cm}\ {\mathrm{s}}^{-1}\right)<\mathrm{T}\mathrm{P}\mathrm{M}\left(2.1\ \mathrm{cm}\ {\mathrm{s}}^{-1}\right)<\mathrm{G}\mathrm{O}\mathrm{M}\ 7.6\left({\mathrm{cm}\ \mathrm{s}}^{-1}\right) $$

According to Marsik et al. (2007), the dry deposition rates of GOM and GEM are much higher in daytime than in night-time. Just as Lindberg et al. (2002), these authors explain this fact by the closure of stomata in night-time. The deposition rate also depends on the type of the surface (Hanson et al. 1995). E.g. Caffrey et al. (1998) determined at the deposition rate of particulate air pollutants on the ground with low vegetation as 3–5 times lower than that in a forest. The depositions are also affected by the weather conditions, air humidity, insolation and atmospheric precipitation.

Feng et al. (2003) and Li et al. (2008) determined the global background air contamination by TGM as 1.5–2.0 ng/m^3^. Slemr et al. (2003) assessed the global background air contamination by TGM on the basis of measurements at 6 stations in the northern hemisphere and 2 in the southern hemisphere, finding that the contamination had grown from 1970, to reach its maximum level in 1980. This was followed by its decrease until 1996 and later the contamination remained at the same level of 1.5–1.7 ng/m^3^ in the northern hemisphere. The GOM concentration in the air in the southern hemisphere was lower as a result of its higher emissions in the northern hemisphere (Pacyna et al. 2006), while its residence time in the air was not long enough for the background contamination to be the same all over the world (Schroeder et al. 1998). Slemr et al. (2008) observed a slight decrease in the TGM concentration at Cape Point at the southern end of Africa, characterising the background contamination for the southern hemisphere, from 1.29 ng/m^3^ in 1996 to 1.19 ng/m^3^ in 2004, while in 1998, Ebinghaus et al. (2002) determined it as 1.26 ng/m^3^. Brunke et al. (2010) reported a further contamination decrease at this measurement point in 2008 to 0.94 ng/m^3^. The mercury concentration in the air in Antarctica was lower than its global background level, amounting in the period from 2007 to 2011 to 0.93 ± 0.19 ng/m^3^ (Pfaffhuber et al. 2012).

Table 6 presents examples of the concentrations of particular mercury species in the air according to different authors. Great caution should be exercised when assessing the results. The measurements were carried out using different methods and more often than not the authors did not determine precisely the mercury species and the measurement duration. In many studies, particularly the earlier ones, the measurements were not subject to quality control.

**Table 6:** Air pollution different forms of mercury, according to various authors

Country/continent	Location	Environment/contamination	Period	TGM	GEM ^2)^	PBM/TPM	RGM/ GOM	Author
				ng/m^3^	ng/m^3^	pg/m^3^	pg/m^3^	
Poland	Lichwin	Rural	2003 summer	1.63	–	110	–	Zielonka et al. 2005
			2004 winter	4.15	–	1050	–	
	Gliwice	Urban	2006–2007	4.1–9.1	–		–	Pyta et al. 2009
	Zabrze	Urban	2006–2007	–	–	61–186	–	
	Southern Baltic	Sea	2008–2009	–	–	0.2–39.9 ^7)^	–	Siudek et al. 2011
				–	–	0.3–151 ^8)^	–	
	Gulf of Gdansk	Coastal	1999–2006	1.0–2.9	–	–	–	Bełdowska et al. 2008
	Gdynia	Coastal	1999–2007	1.2–3.0	–	–	–	
	Hel	Coastal	1997 summer	2.2	–	–	–	Marks and Bełdowska 2001
			1998 winter	1.9	–	–	–	
	Sopot	urban	1999 spring	2.8	–	–	–	Bełdowska et al. 2006a
			1999 autumn	1.5	–	–	–	
			1999 winter	3.3	–	–	–	Bełdowska et al. 2006b
Germany	Waldhof	Background	2009	–	1.66	7.2–9.6	0.73–1.6	Weigelt et al. 2013; Bieser et al. 2014
			2010	–	1.61	6.88	1.29	
			2009	–	1.61	6.42	1.72	
	Wank Mountain	Background	1990 summer	2.97	–	–	–	Slemr and Scheel 1998
			1996 summer/spring	1.82	–	–	–	
	Neuglobosow	Background	1999 autumn	1.98	–	20–74	–	Wängberg et al. 2001
Slovakia	Bratislava	Urban	1996–1997	2.97–3.76	–	200–490	–	Hladíková et al. 2001
	Kosice	Urban		5.13–7.18	–	400–14,800	–	
	Velká Ida	Steel mill		6.37	–	710	–	
	Krompachy	Cu-mill		14.2	–	15,600	–	
	Žiarn.Hronom	Al-mill		8.94	–	150	–	
	Pirvizda	Power station		8.84	–	260	–	
Slovenia	Idrija	Mine-Hg	1970	20,000	–	–	–	Kotnik et al. 2005
			1980	100	–	–	–	
			2004	10	–	–	–	
France	Bordeaux	Urban	2005	–	0.9–2.7	–	–	Pécheyran et al. 2000
		Industrial		–	4.0	–	–	
Irland	Mace Head	Background	1995–2001	1.75	–	–	–	Ebinghaus et al. 2002
			1996	1.80	–	–	–	
			2009	1.40	–	–	–	
	Mace Head	Background	1997–2001	1.76	–	–	–	Kim et al. 2005
	Mace Head	Background	1999 autumn	1.74	–	–	–	Wängberg et al. 2001
Sweden	Rörvik	Background	1980–1989	3.2	–	–	–	Iverfeldt et al. 1995
			1990–1992	2.7	–	–	–	
	Rörvik	Background	1999 autumn	1.54	–	6–31	–	Wängberg et al. 2001
	Aspvreten	Background	–	1.43	–	–	–	
	Geteborg	Urban	2008	–	1.96	12.5	2.53	Li et al. 2008
Great Britain	Wytham Wood	Rural	2007–2008	1.58	–	–	–	Witt et al. 2010
	8 sites—average	Industrial		10.3	–	–	–	
	8 sites—average	Urban		2.0	–	–	–	
	London Westminster Cromwell Road Manchester	Urban	2013 2004 2013 2004 2013 2004	3.28 2.34 3.28 1.84 3.02 1.81	–	–	–	Brown et al. 2015
Spain	Mallorca	Background	1999 winter	2.00	–	40	35–63	Wängberg et al. 2001
	Almadén	Mine-hg	1993–1994	100–50,000	–	–	–	Ferrara et al. 1998
	Munon Cimero	Mine-Hg	2003–2004	170–46,000	–	–	–	Loredo et al. 2006
Italy	Calabria	Background	1999 winter	1.7–2.4	–	12–33	22–42	Wängberg et al. 2001
	Sicilia	Background	1999 summer	1.8–2.0	–	20–40	30–48	Wängberg et al. 2001
	Sicily Etna	Volcano	2004–2007	85–486	–	200–8800	1000–6400	Bagnato et al. 2007
	AbbadiaS. Salvatore	Mine-Hg	1982	8000–243,000	–	–	–	Bellander et al. 1998
	Aeolian Islands	Volcano	2007 winter	172	107	65	–	Zambardi et al. 2009
Mediterranean region	Slovenia	Coastal	2003–2004	4.0	–	–	–	Wängberg et al. 2008
	Italy			1.75	–	–	2.6–2.7	
	Israel			–	–	–	2.2	
	Spain			1.6–2.1	–	–	0.2–7.0	
Korea	Seoul	Urban	1987–1988	14.4	–	–	–	Kim and Kim 2002
			1999–2000	5.4				
			2012–2013	2.3				Kim et al. 2016
South Korea	Seoul	Urban	2005 spring	3.11	–	–	–	Kim et al. 2009
			2005 summer	2.59	–	17.7	21.5	
			2005 autumn	3.16	–	26.7	31.1	
			2005 winter	3.89	–	25.0	26.2	
China	Tibet	Background	2006 winter	3.98	–	–	–	Fu et al. 2008
	Beijing	City centre	1998 winter	7.9–8.6	–	–	–	Liu et al. 2002
			1998 summer	13.8–11.4	–	–	–	
		Urban	1998 winter	11.6–34.9	–	–	–	
			1998 summer	8.1–12.1	–	–	–	
		Industrial	1998 winter	5.3	–	–	–	
			1998 summer	7.3–9.0	–	–	–	
		Suburban	1998 winter	6.1–12.4	–	–	–	
	Huairou	Rural	1998 winter	0.7–1.0	–	–	–	
			1998 summer	1.6–1.7	–	–	–	
	Wucguan	Mine-Hg	2005 summer	40–40,000	–	–	–	Lin et al. 2012
		Rural		18–28	–	–	–	
	Xunyang	Mine-Hg	2011	7.4–410	–	–	–	Qiu et al. 2012
	Guizhou	Zn-smelter	2002	30–15,090	–	–	–	Feng et al. 2006
		Residential area	2002	10–50	–	–	–	
Taiwan	Hung-Kung	Industrial	2010–2011	6.14	–	2.3	332	Huang et al. 2012
	Labs	Background	2006 spring	–	1.70	2.6	10.4	Sheu et al. 2010
			2006 summer	–	1.33	1.1	8.0	
			2006 autumn	–	2.01	1.7	15.8	
			2007 winter	–	1.98	4.5	15.2	
RPA	Cape Point	Background	1995–1999	1.23	–	–	–	Baker et al. 2002
			2011	0.92	–	–	–	Slemr et al. 2015
			2013	1.05	–	–	–	
Southern Atlantic	2–54°S	Background	1977–2001	0.24–2.44	–	–	–	Temme et al. 2003
Northern Atlantic	5–67°N			1.00–3.73	–	–	–	
Canada	Egbert	Background	1997–2001	1.69	–	–	–	Kim et al. 2005
	Point Peter			1.93	–	–	–	
	Burnt Island			1.58	–	–	–	
	Arctic	Background	1997–2001	1.55	–	–	–	Kim et al. 2005
	Bay St. Francois	Background	2002	1.40	1.38	6.44	3.63	Poissant et al. 2004
	St. Anicet Quebec	Background	2003	1.65	–	–	–	Poissant et al. 2005
	Flin Flon	Cu-smelter	2011	–	2.06	10.4	3.4	Cole et al. 2014
	Kejimkujik	Background	2002–2009	–	1.34	4.2	0.5	
USA	Oregon	Background	2004 spring	1.69–1.84	–	–	–	Weiss-Penzias et al. 2006
			2005 summer	1.54	5.2	43	–	Swartzendruber et al. 2006
	Lake Michigan	Background	1997–1999	1.60	–	12–70	–	Landis et al. 2002
			1995–2005	1.58	–	6–133	–	
	Detroit	Urban	1999–2002	–	1.09–15.8	21–30	18–29	Lynam and Keeler 2005
	Detroit	Urban	2004	–	2.47	16	18.	Liu et al. 2010
	Dexter	Suburban		–	1.59	3.8	6.1	
	Connecticut	Urban	1997–1999	2.19–2.69	–	9.7–16.2	–	Nadim et al. 2001
		Rural		1.60–1.74	–	6.1–7.4	–	
	Stan N. York	Urban	2001–2002 summer	1.83–3.02	–	–	4.2–6.0	Han et al. 2004
	Adirondacks	Forest	2006–2007	–	1.4	3.2	1.8	Choi et al. 2008
	New York	Urban	2000	–	–	–	–	Carpi and Chen 2002
		Manhattan		3.30–4.56	–	–	–	
		Brooklyn		3.70	–	–	–	
		Queens		2.69	–	–	–	
	San Francisco	Cement factory	2005–2008	–	2.20	81	25.2	Rothenberg et al. 2010
		Urban		–	2.28	3.2	2.9	
		Rural		–	2.37	8.0	14.5	
Kolumbia	Remedios	ASMG	2010	–	–	–	–	Cordy et al. 2011
		Rural		1–20	–	–	–	
		Urban		500–10,000	–	–	–	
	Segovia	Urban		100–1500	–	–	–	
	Zaragosa	Urban		40–3000	–	–	–	
Surinam	Paramaribo	Background	2006–2007	1.4	–	–	–	Wip et al. 2013
		Urban		5.6 (max 109)	–	–	–	
Galapagos		Background	2011	1.05	–	–	–	Slemr et al. 2015
Philippines	Palawan	Mine-Hg closed	2002	64.9	–	–	–	Maramba et al. 2006
Australia	Sydney	Urban	2006–2007	–	1.5–1.1	1.5–8.6	–	Dutt et al. 2009
	Cape Grim	Background	2011	0.96	–	–	–	Slemr et al. 2015
Arctic	Ellesmere Island Spitsbergen	Background	2000–2009	1.50 1.57	–	–	–	Cole et al. 2013
Antarctica	Troll	Background	2011 + 2012 + 2013	1.03 1.05 0.97	–	–	–	Slemr et al. 2015

An analysis of the results given in the table indicates a large differentiation of the levels of air contamination by mercury. The highest TGM levels were determined in the areas of mercury mines: in Italy, at Abbadia San Salvatore in 1982, 8.000–243.000 ng/m^3^ (Bellander et al. 1998); in Spain, at the Almadén Mine in 1993–1994, 100–50.000 ng/m^3^ (Ferrara et al. 1998) and at Munon Chimero in 2003–2004, 170–4.600 ng/m^3^ (Loredo et al. 2006), and in Slovenia, at the Idrija Mine, 2.000 ng/m^3^ (Kotnik et al. 2005). The areas where gold was mined and processed at a small scale (ASGM areas) were also characterised by high concentrations, e.g. in Columbia, in the area of the town of Remedios, in 2009–2010, 10,000 ng/m^3^ as a maximum (Cordy et al. 2011), and in China, in Tongguan, 33,000 ng/m^3^ as a maximum and in Wuchuan ng/m^3^, 40,000 (Lin et al. 2012).

High levels of air contamination by mercury contained in particulate matter (TPM/PBM) and in gaseous oxidized form (RGM/GOM) are characteristic of areas adjacent to active volcanoes. For example, in the vicinity of the Etna volcano in Sicilia, in the period from 2004 to 2007, the PBM/TPM concentrations amounted to 200–8800 pg/m^3^ and those of RGM/GOM were 1000–6400 pg/m^3^, with the TGM concentrations varying between 85 and 486 ng/m^3^ (Bagnato et al. 2007). Varekamp and Buseck (1986) determined the TGM concentrations in 1971 in the vicinity of the Kilauea volcano on Hawaii to vary between 274 and 1031 ng/m^3^.

In industrial areas, the mercury contamination in the air exceeded, as a rule, 5 ng/m^3^, whereas in cities it tended to be lower than 4 ng/m^3^. Relatively high TGM levels were found in Kosice in Slovakia, varying between 5.13 and 7.17 ng/m^3^ (Hladíková et al. 2001), in Beijing in China, varying between 5.3 and 34.9 ng/m^3^ (Liu et al. 2002), and on Manhattan in New York City, varying between 3.30 and 4.56 ng/m^3^ (Carpi and Chen 2002).

## Conclusions

Air contamination by mercury continues to be one of the most important problems of the contemporary world. The reason for this is the high toxicity of mercury for man. At the International Conference on Heavy Metals in the Environment (ICHMET-15 2010), as many as 85 papers and posters concerned mercury.

The following general conclusions can be drawn from a review of the literature:

- The assessment of mercury emissions into the air poses serious methodological problems. In estimating them, state institutions mainly focus on inventories of their sources, while international organisations apply different models, based on emission factors and statistical data on the industrial manufacture of products using mercury and the consumption of mercury-containing raw materials.
- It is particularly difficult to distinguish between natural and anthropogenic emissions and re-emissions from lands and oceans, including past emissions. The estimated emission levels given by particular sources are different by as much 100%.
- Until the early twentieth century, the largest emission source had been the silver and gold mining called “a gold rush”. In recent years, the largest emission sources included fuel combustion, mainly that of coal, and “artisanal and small-scale gold mining” (ASGM).
- Until the Second World War, the highest emissions came from North America and Europe. At present, South and South-East Asia strongly dominate, accounting for 45% of the global emissions.
- The emissions of natural origin and re-emissions are estimated at 45–66% of the global emissions, with the largest part of emissions originating in the oceans.
- Forecasts on the future emission levels are not unambiguous. The emission levels will depend on the effectiveness of international conventions (the Minamata Convention, Selin 2014) imposing limitations on mercury emissions. However, most forecasts do not provide for reductions in emissions.
- Mercury occurs in the air in different species: gaseous elemental mercury (Hg^0^—GEM), gaseous mercury in oxidized form (Hg^II^—GOM), mercury in particulate matter w (Hg_p_—TPM) and mercury in organic compounds (MeHg). Depending on the weather conditions and the presence of other pollutants, mercury undergoes numerous transformations.
- The GEM species represents about 95% of its total mass in the air and can be transported between continents, while its residence time in the air is estimated at 6 to 18 months. In the case of the GOM and TPM species, the residence times are estimated at hours and days.
- The highest mercury concentrations in the air can be found in the areas of mercury mines and those of artisanal and small-scale gold mining (ASGM). Since 1980 when it reached its maximum the global background mercury concentration in the air has remained at a relatively constant level.
